# Compliance with The RSV Immunoprophylaxis Dosing Schedule in The Polish Registry for Palivizumab (2008-2014)

**DOI:** 10.34763/devperiodmed.20182204.308314

**Published:** 2019-01-14

**Authors:** Róża Borecka, Ryszard Lauterbach

**Affiliations:** 1 Pediatric Unit, Independent Public Health Care Center, Myślenice, Poland; 2 Department of Neonatology, Jagiellonian University Medical College, Kraków, Poland

**Keywords:** respiratory syncytial virus infections, palivizumab, compliance, prophylaxis, zakażenia syncytialnym wirusem oddechowym, paliwizumab, przestrzeganie zaleceń, profilaktyka

## Abstract

**Background:**

*Respiratory syncytial virus infection causes respiratory diseases in about 90% of the children under 2 years of age. Currently the only way to prevent infection is through immunoprophylaxis based on palivizumab*.

**Aim:**

*The aim of the study was to assess compliance with the recommended prophylaxis regimen in children qualified for the Polish National Programme for Respiratory Syncytial Virus Immunoprophylaxis over six consecutive virus seasons (2008-2014)*.

**Material and methods:**

*A retrospective analysis of data obtained from a multicentre, non-interventional observational study was performed. The prevention programme included 3,780 children aged 4 weeks to 2 years. The analysis included: the course of the neonatal period, clinical features at the time of inclusion in the programme, the immunisation course, and adherence to the palivizumab dosing schedule*.

**Results:**

*During the programme, the children received an average of 3.8 (range 1-5) injections. The highest mean number of injections was recorded in the 2013/14 season (4.3±1), and the lowest in the 2009/10 season (2.7±0.8). Overall, 3,084 children (81.7%) received all of the expected doses, while 2,352 (62.2%) children received injections within the appropriate interdose interval. The probability of noncompliance was higher for males. None of the other demographic, social, or clinical factors seemed to impact compliance*.

**Conclusions:**

*Compliance with the monthly dosing schedule of palivizumab is key to achieving the proper immunoprophylaxis efficacy. Education regarding the consequences of non-compliance with the regime and increased doctor-parent communication is recommended in future*.

## Streszczenie

**Wstęp:**

*Zakażenie wirusem syncytium nabłonka oddechowego jest przyczyną chorób dróg oddechowych u ok. 90% dzieci poniżej 2. roku życia. Jedyną możliwością zapobiegania zakażeniom tym wirusem jest immunoprofilaktyka prowadzona w oparciu o podaż paliwizumabu*.

**Cel:**

*Celem badania była ocena przestrzegania zaleceń i wyników zdrowotnych u dzieci zakwalifikowanych do Ogólnopolskiego Programu Zapobiegania RSV w ciągu sześciu sezonów zakażeń wirusem syncytium (2008-2014)*.

**Materiał i metody:**

*Analizę retrospektywną przeprowadzono na podstawie danych uzyskanych z wieloośrodkowego, nieinterwencyjnego badania obserwacyjnego. Programem profilaktyki objęto 3780 dzieci w wieku od 4 tygodni do 2 lat. Analiza obejmowała: przebieg okresu noworodkowego, cechy kliniczne w momencie włączania do programu, przebieg i przestrzeganie zaleceń dawkowania paliwizumabu*.

**Wyniki:**

*W trakcie programu dzieci otrzymały średnio 3,8 iniekcji (zakres od 1 do 5 dawek). Najwyższą średnią iniekcji odnotowano w sezonie 2013/14 (4,3±1), a najniższą w sezonie 2009/10 (2,7±0,8). 3084 dzieci (81,7%) otrzymało wszystkie oczekiwane dawki, natomiast 2352 (62,2%) dzieci otrzymało iniekcje zachowując reżim czasowy. Prawdopodobieństwo nieprzestrzegania zaleceń było większe w przypadku dzieci płci męskiej*.

**Wnioski:**

*Przestrzeganie miesięcznego schematu dawkowania paliwizumabu jest kluczowe dla uzyskania właściwej skuteczności immunoprofilaktyki. Badanie wskazuje na potrzebę edukacji w zakresie skutków wynikających z nieprzestrzegania reżimu i zalecenie wzmożonej komunikacji lekarz-rodzic*.

## Introduction

Respiratory syncytial virus (RSV) is a highly contagious pathogen that infects over 90% of all children by two years of age. In healthy full-term infants in the second half-year of life and in older children, RSV infection may be a mild, self-limiting disease. However, in some cases, especially infants under 6 months of age and high-risk children, symptoms of lower respiratory tract infections (LRTIs) develop in the form of bronchitis, severe bronchiolitis or pneumonia with respiratory failure [1, 2, 3]. In these patients it may be necessary not only to be hospitalised in the intensive care unit, but also to use mechanical ventilation. Indeed, data on mortality in the course of infection indicate that despite advances in neonatal intensive care, RSV infection is a significant cause of infant deaths [4, 5]. An increased risk of a severe course of infection requiring hospitalisation and mechanical ventilation occurs primarily in children born prematurely, in infants with bronchopulmonary dysplasia (BPD) and in those with a hemodynamically significant congenital heart defect.

Due to the lack of effective methods for treating bronchiolitis caused by RSV, the prevention of infection plays a fundamental role. So far, no effective RSV vaccine has been developed and the only option to prevent RSV infection is immunoprophylaxis with palivizumab. Palivizumab is a humanised monoclonal immunoglobulin G class antibody that binds to the F (fusion) glycoprotein of the RSV, preventing its penetration into the airway epithelium. The immunoglobulin is given once a month at a dose of 15 mg/kg of body weight by intramuscular injection. Prophylaxis is carried out during the high-risk season, which in Poland occurs from October to April.

Since 2008, the Polish National Programme for RSV Immunoprophylaxis (OPZRSV) has been conducted in our country, under which all patients included in preventive treatment are closely monitored. Data on demographics, clinical outcomes and results of prophylaxis with palivizumab in infants are recorded. Published results of OPZRSV confirm the efficacy of the prophylaxis conducted [6]. Since the effectiveness of immunoprophylaxis depends on the compliance with the monthly dosing schedule, it is necessary to monitor adherence with the recommendations and to ascertain any factors that may affect it. The data published so far on adherence to the recommendations among children from the OPZRSV registry indicated the need for further research to determine new factors that may interfere with palivizumab use in the Polish population [7].

### Objectives

The aim of this study was to assess compliance with the recommendations of all children enrolled into the OPZRSV over six consecutive RSV seasons (2008–2014) and to compare the data obtained with corresponding published data from other countries.

## Material and methods

The study includes children enrolled into the OPZRSV who received at least one palivizumab dose financed by the National Health Fund (NFZ) over six consecutive RSV seasons (season I: 2008/09, season II: 2009/10, season III: 2010/11, season IV: 2011/12, season V: 2012/13 and season VI: 2013/14). The inclusion criteria were not identical for all the periods of prophylaxis and were subject to changes depending on updates to guidelines introduced by the Ministry of Health and the NFZ.

In the 2008/09 season, prophylaxis included infants with BPD who fulfilled one of the following conditions:

- preterm birth in 2008 and gestational age of ≤30 weeks;
- preterm birth in 2007 and gestational age of ≤26 weeks;
- severe BPD requiring ongoing medical treatment.

From 2009 to 2013 (seasons II–V), prophylaxis was also funded by the NFZ, but more restricted inclusion criteria were applied. The programme enrolled children with BPD who met one of the following criteria:

- gestational age of <30 weeks and <3 months of age at the beginning of the RSV infection season (born after August 1);
- gestational age of <28 weeks and <6 months of age at the beginning of the RSV infection season (born after May 1).

In season VI, prophylaxis was implemented in children who at the time of initiation of the immunisation did not complete their first year of life and met one of the following criteria:

- gestational age of ≤28 weeks, 0 days;
- BPD.

BPD was defined as the necessity to use oxygen therapy with oxygen concentration above 21% for up to 28 days of life.

The exclusion criteria were: hypersensitivity to the active substance (palivizumab) or to any of the constituents present in the preparation; contraindications for passive immunisation; and lack of parental or legal guardian consent to the administration of immunoglobulin.

The study was retrospective, observational, multicentre, and non-interventional. During the six seasons, the study included 32 neonatal centres throughout the country. For each child enrolled for the prophylactic supply of palivizumab, demographic data were collected, and the neonatal course and details of palivizumab administration were analysed.

### Statistical analysis

The analysis of the data included assessment of the following factors: the neonatal course, clinical features at the time of inclusion in the programme, the use of palivizumab and adherence to the recommendations of the monthly dosing schedule.

Compliance with the recommendations was assessed in two ways: comparing the actual number of received doses with the expected number of doses; and the interdose interval.

The expected number of palivizumab doses was calculated by assuming monthly injections throughout the high-risk RSV infection season. It was assumed that children who received the first dose of palivizumab in October, November or December receive a total of five doses, and those who received the first dose in March should receive a total of two doses of immunoglobulin. Any child who received all the expected doses, was considered compliant. Interdose intervals of 30 ± 5 days were considered compliant. However, as an interval of 20 ± 5 days between the first and the second dose was likely to result in higher trough levels after the first dose, potentially providing better protection against RSV, an interval of 16–35 days between the first and the second injection was considered to be compliant.

In the descriptive characteristics for categorical variables, the number of observations and the percentage of occurrences were taken into account. Categorical variables were compared by the Chi-square test or Fisher’s exact test (for variables with the size of at least one category smaller than 5). Continuous variables were first analysed using the Shapiro-Wilk test, then depending on the result obtained, the mean and standard deviation (SD) were reported for variables with normal distribution, while for variables with a non-normal distribution, the median was presented with quantiles of 25% and 75% (Q1 and Q3). Variables with normal distribution were compared with the Student’s t test, otherwise, the Mann-Whitney test was used.

In the analysis of the impact of the considered factors on compliance, an odds ratio (OR) with a 95% confidence interval (CI) was also reported. In the case of continuous variables, the observations were divided into two groups, with the value of the variable above and below the median of this variable for the whole group, respectively.

The statistical analysis was carried out using the statistical package R, version 3.1.2. In all analyses, the 0.05 significance level was assumed. Methods for filling in data gaps were not used.

## Results

### Patients’ characteristics

The prevention programme enrolled a total of 3,780 infants, and ranged from 464 to 995 children per individual season. Patient characteristics including data from the perinatal period and chronological age at the start of the immunisation are given in Table I. The study included 2.013 (53.3%) boys and 1.767 (46.7%) girls. In all the seasons, except for 2010/11, male infants accounted for over 50% of the population covered by prophylaxis. The average gestational age was 26.8.±.1.7 weeks, and the average birth weight was 960.9.±.268.1 g.

### The course of prophylaxis

The prevention programme was started in children aged from 4 weeks to 2 years, and the average chronological age at the start of the immunisation was 162 days. A total of 14.275 doses of palivizumab were administered. Children received an average of 3.8 injections of palivizumab, ranging from 1 to 5 doses. The average number of injections was the highest in the 2013/14 RSV season, and the lowest in the 2009/10 season (4.3±1.0 vs 2.7±0.8; tab. II).

### Adverse events

Adverse events occurred after 392 doses of palivizumab (2.7%, 392/14275). Anxiety was most common (1.2%), while the other adverse effects were less frequent and had a similar incidence. These events were generally reported to be mild and of short duration. No child had the drug discontinued for a related adverse event.

### Compliance with recommended dosing

**Fig. 1:**
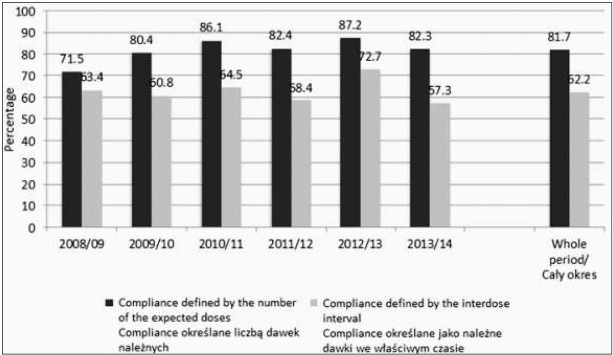
Compliance with the recommended dosage year by year. *Ryc. 1. Przestrzeganie zalecanego dawkowania rok po roku*.

During the six seasons of the RSV prophylaxis programme, a group of 3,084 children (81.7%; incomplete data on the dates of immunisation in 13 children) received all the expected doses, while only 2,352 (62.2%) children received subsequent injections within the appropriate interdose interval (Fig. 1).

The best compliance was observed in the 2013/14 season, both in terms of compliance defined as the administration of all the expected doses, as well as compliance defined as the administration of subsequent injections within the appropriate interdose intervals. The lowest compliance took place in the first two seasons of immunisation. A significant correlation was observed between compliance and the sex of infants, with a higher proportion of male children in the group of non-compliant patients (tab. III). There was also higher probability of non-compliance in the group of children who were older at the start of the immunisation; however, for compliance defined as the supply of the expected doses within the appropriate interdose interval, no such relationship was observed.

**Tabela I:** Charakterystyka populacji badanej w podziale na sezony. Table I. Characteristics of the study population by RSV season.

*Characteristics Charakterystyka*	2008/09	2009/10	2010/11	2011/12	2012/13	2013/14	** Total Ogółem
Number of infants included in the study, n *Liczba niemowląt* *włączonych do badania, n*	557	464	582	556	626	995	3,780
Male infants, n (%) *Płeć męska*, n *(%)*	302 (54.2)	250 (53.9)	286 (49.1)	282 (50.7)	369 (58.9)	524 (52.7)	2,013 (53.3)
Gestational age, mean (SD), weeks *Wiek ciążowy, średnia (SD), tyg*.	26.6 (2.0)	26.6 (1.4)	26.6 (1.5)	26.7 (1.5)	26.7 (1.6)	27.2 (2.0)	26.8 (1.7)
Birth weight, mean (SD), g *Urodzeniowa masa ciała, średnia (SD), g*	941.5 (280.5)	931.5 (237.8)	917.9 (251.6)	953.2 (232.5)	961.8 (240.1)	1014.5 (307.8)	960.9 (268.1)
Chronological age at the start of immunisation, mean (SD), days *Wiek chronologiczny na początku immunizacji, średnia (SD), dni*	271.9 (146.3)	163.3 (70.3)	123.6 (44.9)	108.5 (43.5)	126.2 (73.7)	174.6 (101.9)	162.3 (103.6)

**Tabela II:** Przebieg immunizacji. Table II. The course of immunisation.

	2008/09	2009/10	2010/11	2011/12	2012/13	2013/14	Total *Ogółem*
Number of children *Liczba dzieci*	557	464	582	556	626	995	3,780
Total number of doses *Ogólna* *liczba* *dawek*	2,001	1,240	2,154	2,198	2,367	4,315	14,275
Number of doses/child *Dawki/dziecko*	3.6	2.7	3.7	3.9	3.8	4.3	3.8
Number of adverse events *Liczba zdarzeń niepożądanych*	72	36	68	54	61	101	392
Number of adverse events/number of doses *Liczba* *zdarzeń* *niepożądanych/liczba dawek*	0.04	0.03	0.03	0.02	0.03	0.02	0.03

There were no significant differences concerning gestational age and birth weight between the compliant and non-compliant groups, when evaluated by both definitions (tab. III).

## Discussion

Compliance with the monthly dosing schedule of palivizumab is key to maintaining its therapeutic concentration and effective inhibition of RSV replication. Insufficient compliance significantly affects the effectiveness of immunoprophylaxis and may increase the risk of severe LRTIs caused by RSV [8]. For this reason, it is necessary to monitor the course of immunisation and identify factors affecting cooperation with the doctor and compliance with the dosage regimen.

The data from the six OPZRSV seasons presented in our study reveal that 81.7% of the children received all the expected doses, and 62.2% of the children received all their injections within the appropriate interdose interval. These results are similar to data from other published registers of large-scale national prevention programmes, which included from several thousands to over 10,000 patients. For example, over nine seasons (from 2005 to 2014), The Canadian Registry of Palivizumab (CARESS) estimated that 81.2% of children received all the expected doses, and 60.9% of infants received all injections within the appropriate interdose interval [8]. Likewise, in two studies presenting five seasons of the Palivizumab Outcomes Registry (i.e., from 1998 to 1999 and from 2000 to 2004), 79.8-86.0% of the children were compliant in terms of receiving all the expected doses [9, 10]. Data regarding the administration of all injections within the appropriate interdose interval were published for only four seasons (2000-2004), and amounted to 59.3% [9, 10]. Finally, based on six seasons of the German Palivizumab Registry (1999-2005), 73% of the patients received all doses within the appropriate interdose interval [11].

**Tabela III:** Czynniki wpływające na przestrzeganie zaleceń immunoprofilaktyki RSV 2008-2014. Table III. Factors affecting RSV immunoprophylaxis compliance from 2008 to 2014.

Characteristics Charakterystyka		Compliance defined by the number of the expected doses *Compliance określane liczbą dawek należnych*			Compliance defined by the interdose interval *Compliance określane jako należne dawki we właściwym czasie*		
		Compliant *zgodnie z zaleceniami n=3084*	Non-compliant *niezgodnie z zaleceniami n=683*	P-value *Wartość p*	Compliant *zgodnie z zaleceniami n = 2352*	Non-compliant *niezgodnie z zaleceniami n = 1415*	P-value *Wartość p*
Sex *Płeć* n (%)	M	1,610	396	**0.007**	1,228	778	0.106
	M	(80.3)	(19.7)		61.2	(38.8)	
	F	1,474	287		1,124	637	
	K	(83.7)	(16.3)		(63.8)	(36.2)	
Duration of gestation below the median Czas trwania ciąży poniżej mediany n (%)	Yes	2,080	477	0.271	1,584	973	0.393
	Tak	(81.3)	(18.7)		(61.9)	(38.1)	
	No	998	206		764	440	
	Nie	(82.9)	(17.1)		(63.5)	(36.5)	
Chronological age below the median Wiek chronologiczny poniżej mediany n (%)	Yes	1,499	293	**0.003**	1,121	671	0.990
	Tak	(83.6)	(16.4)		(62.6)	(37.4)	
	No	1,422	361		1,114	669	
	Nie	(79.8)	(20.2)		(62.5)	(37.5)	
Birth weight below the median Urodzeniowa masa ciała poniżej mediany n (%) **	Yes	1,534	351	0.460	1,165	720	0.442
	Tak	(81.4)	(18.6)		(61.8)	(38.2)	
	No	1,550	332		1,187	695	
	Nie	(82.4)	(17.6)		(63.1)	(36.9)	

Our results also indicate that compliance changes along with the duration of OPZRSV, with the lowest compliance (as defined by the administration of all the expected doses) occurring in the first two seasons of the immunisation programme. A higher compliance in the subsequent years of the programme was also highlighted in the Canadian registry (CARESS), which may reflect an increase in the knowledge and experience of health professionals with respect to running the OPZRSV [8].

A higher probability of adherence was also observed in children who were younger at the start of the immunisation programme, which is in line with the results of the Italian cohort study and the Canadian registry [8, 12]. Since the predisposition to developing a severe LRTI caused by RSV is inversely proportional to the chronological age, the awareness of this threat among parents seems to be a factor conducive to compliance. However, in contrast to Italian researchers, we did not find a higher rate of adherence to the dosing regimen (as assessed by any method) among children with lower birth weight.

We did however, find the probability of non-compliance to be higher in male children. This is worrying because boys have shorter and narrower airways and are therefore more likely to develop bronchial obstruction in the case of RSV infection [13, 14]. In addition, male sex is a risk factor for hospitalisation associated with RSV infection in young children [15]. However, this knowledge is not common among parents. It seems that, on the contrary, male children can be perceived by the family as stronger and thus not requiring prophylaxis.

There are a few limitations of our study. First, this was an observational study without a comparative placebo arm. Second, administrative factors could adversely affect compliance with the dosage regimen. An example may be a situation in which an infant did not receive palivizumab during hospitalisation because the centre did not have accreditation to conduct the OPZRSV.

## Conclusions

Our data from the OPZRSV are similar to prevention programmes conducted in other countries in terms of compliance with the monthly injection regimen during the RSV season. However, compliance rates do not reach >90%, as previously reported in randomised clinical trials. This difference between registries and randomised trials seems to be due to the reduction of compliance rates over time, that is, from the first injection to the last injection during the RSV season.

Educating parents about the risks associated with RSV infection, including the risk factors for a more severe disease course, along with the benefits of immunoprophylaxis is crucial for improving compliance with the palivizumab dosing schedule [12, 16, 17, 18]. Parents should be informed that this is passive immunisation and not vaccination, which is why a monthly supply of immunoprophylaxis over the RSV season is necessary for it to be effective.

In the current and previously published studies presenting data from the OPZRSV, there are no demographic or neonatal features affecting compliance except for sex and chronological age at the time of the first dose administration. Therefore, further actions aimed at optimising the prevention programme should be directed to the entire population covered by the prevention programme, taking into account the fact that the parents of boys should be aware of the risk related to their child’s sex.
